# Decoding allergy in vitro: Challenges and clinical use of humoral and cellular methods 

**DOI:** 10.5414/ALX02600E

**Published:** 2026-05-05

**Authors:** Bettina Wedi, Timo Buhl

**Affiliations:** 1 Department of Dermatology and Allergy, Comprehensive Allergy Center, Hannover Medical School, Hanover, and; 2 Department of Dermatology, Venereology and Allergology, University Medical Center Göttingen, Göttingen, Germany

**Keywords:** in vitro diagnostics, allergy testing, specific IgE, component-resolved diagnostics, basophil activation test

## Abstract

In vitro assays are essential tools in diagnosing IgE-mediated allergic diseases and complement clinical history and skin testing across food, inhalant, venom, and drug allergies. This methodological review is intended for clinicians seeking a more detailed understanding of current laboratory-based allergy diagnostics. It summarizes current humoral and cellular diagnostic methods, including total IgE, singleplex and component-resolved specific IgE testing, multiplex IgE platforms, and functional cellular assays such as the basophil activation test and T-cell–based approaches. Humoral assays remain the quantitative foundation of molecular allergy diagnostics, while multiplex arrays broaden diagnostic scope and map sensitization profiles, although platform variability requires cautious interpretation. Cellular assays may add functional information in selected constellations but remain technically demanding, insufficiently standardized, and are best regarded as adjunctive tools in specialized or research settings, with evidence largely derived from selected cohorts. Additional areas – immunotherapy monitoring, mast cell disorders, contact allergy, and oncology – illustrate the broader methodological spectrum of in vitro methods. Persistent unmet needs include harmonization, validated reference standards, and robust interpretive frameworks. Disease-specific applications are discussed in companion articles in this issue.

## Introduction and scope of the article 

In vitro allergy diagnostics play an important role in the evaluation of IgE-mediated allergic diseases. As the cornerstone of objective allergen-specific diagnosis, in vitro testing complements clinical history and skin testing, offering a safe, reproducible, and standardized approach – particularly in patients with contraindications to skin testing or severe dermatologic conditions. This review provides clinicians with a methodologically focused overview of current in vitro diagnostic strategies and their practical interpretation in routine allergy work-up. It outlines key strengths and limitations, including quantitative readouts, variable sensitivity, clinically irrelevant cross-reactivities, and challenges in standardization. Results must be interpreted within the clinical context and integrated with other diagnostic tools. The focus is on methodological principles and interpretation, whereas disease-specific applications are addressed in companion articles in this issue. 

A central distinction is between humoral (IgE-based) and cellular approaches (e.g., basophil activation tests, T-cell assays). While humoral diagnostics remain the basis of routine testing, cellular assays may provide functional information in selected cases but have limited standardization and mainly adjunctive clinical applicability. A structured, evidence-based integration of these methods is required to support clinical decision-making. This review is primarily intended for clinicians and laboratory specialists experienced in allergy diagnostics. 

## Humoral in vitro assays 

Humoral assays quantify circulating immunoglobulins – principally total IgE (tIgE) and allergen-specific IgE (sIgE) – to document sensitization and to support clinical decisions when interpreted with exposure history, phenotype, and, where relevant, challenge outcomes [1, 2, 3, 4]. A concise matrix of analytical and clinical features of humoral singleplex assays is provided in Table 1. 

### Total IgE 

tIgE reflects overall type-I atopic status but has modest standalone diagnostic value. Very low tIgE (e.g., values close to the lower detection limit, typically < 10 – 20 kU/L) reduces the likelihood of detectable sIgE; high tIgE (e.g., several hundred kU/L or higher) increases it but does not confirm clinical allergy [1]. Reference intervals are age- and assay-dependent; laboratories should use method-specific reference intervals validated locally against method-specific reference populations, ideally including clinically characterized populations [5, 6]. Despite traceability to WHO IgE standards (75/502; 11/234), between-method bias persists; tIgE is best used as a contextual covariate, not a binary trigger [7, 8]. In ABPA, markedly elevated tIgE (often > 1,000 kU/L), together with *Aspergillus*-specific IgE and IgG, contributes to diagnosis and monitoring [9]. 

Practical roles: (i) Pre-test framing: low tIgE lowers the prior probability of sensitization; (ii) interpretation of low-titer sIgE; (iii) ABPA algorithms and selected longitudinal use [1, 9]. 

### Allergen-specific IgE (singleplex) 

Singleplex assays quantify sIgE against defined extracts or molecular components and remain the workhorse across major clinical indications (foods, aeroallergens, venoms, selected drugs) [1, 10]. Modern analytic sensitivity reaches < 0.10 kU_A_/L; the historical 0.35 kU_A_/L threshold reflects earlier assay limits and is not a universal clinical threshold [11]. Low-level sIgE (0.10 – 0.35 kU_A_/L) may be clinically relevant in high pre-test probability scenarios (such as recent systemic reactions, concordant exposure), but requires integration with history, tIgE, and phenotype [2, 11]. 

**Predictive values and decision points**

For several foods, higher sIgE titers correlate with oral food challenge outcomes and permit decision points that may reduce challenges; values are allergen- and population-specific [12, 13, 14]. Cut-points derived on one platform or cohort should not be transferred without validation in local patient populations and clinical settings, ideally against outcome-based reference standards [15]. For inhalant allergens, quantitative sIgE supports diagnosis and AIT planning when aligned with exposure and symptoms; for venoms, extract-based sIgE documents sensitization before and during venom immunotherapy [1, 16]. 

**Inter-platform comparability**

sIgE values correlate across commercial systems but show systematic biases; longitudinal monitoring and application of literature cut-offs should be restricted to a single platform [17, 18, 19]. Reporting should include method, allergen source, and units. 

**Interpreting low titers and ratios**

The sIgE/tIgE ratio has been explored to refine interpretation in selected contexts; evidence is heterogeneous, and routine use – particularly for food allergy diagnosis – is not supported by current guidelines [20, 21]. It may be considered in selected cases with high pre-test probability but should not be used in isolation. In low pre-test probability, isolated low-titer sIgE often reflects clinically irrelevant sensitization. Interpretation should also consider age, timing of testing, and potential medication effects (e.g., anti-IgE therapy), as these factors may influence detectability and clinical relevance. 

**IgG/IgG4**

Food-specific IgG/IgG4 has no diagnostic value for food allergy and is not recommended by professional societies; allergen-specific IgG retains a role in selected entities (e.g., *Aspergillus*-IgG in ABPA) but does not replace IgE-based diagnostics [9, 22, 23]. In the context of allergen immunotherapy, increases in allergen-specific IgG or IgG4 reflect immunological changes but do not reliably predict clinical outcomes. 

### Component-resolved diagnostics (singleplex) 

Component-resolved diagnostics (CRD) quantify sIgE to defined molecules (e.g., storage proteins, LTPs, PR-10 proteins and profilins in foods; major venom allergens such as Api m/Ves v proteins in insect venom allergy). CRD improves discrimination between primary sensitization and cross-reactivity, supports risk stratification (e.g., stable storage proteins vs. labile PR-10), and refines AIT selection [3, 4, 16]. Minor cross-reactive components such as profilins and polcalcins should also be considered in this context. Decision thresholds remain molecule- and cohort-specific; clinical correlation is mandatory [2]. 

### Methodological and practical considerations 

Common analytical and interpretive pitfalls are summarized in Table 2. 

**Calibration and harmonization**

WHO IgE standards underpin calibration, yet harmonization across platforms remains incomplete for both tIgE and sIgE [7]. External quality assessment (EQA) shows good but variable inter-laboratory agreement, supporting the need for ongoing proficiency testing and local verification of RIs and decision thresholds [8, 24, 25]. 

**CCD interference**

IgE to CCDs may cause clinically irrelevant positive results, especially with plant or insect extracts, affecting aeroallergen, food, and some venom testing. CCD blockers and targeted component testing can mitigate this and should be considered in low-titer polyreactivity without clinical correlation [26, 27, 28]. 

**Aeroallergens**

For rhinitis or asthma due to pollens, mites, animal dander, and molds, singleplex sIgE supports diagnosis and AIT planning when aligned with exposure and phenotype. If history and test results diverge, reassess extract quality, consider CCDs, and weigh pre-test probability before acting on low-titer findings [1, 15, 28]. 

**Insect venom allergy**

Venom sIgE documents sensitization and, combined with history and (when needed) skin testing, supports decisions on immunotherapy. CRD can clarify cross-sensitization in cases of double positivity (e.g., honeybee vs. vespid), although immunotherapy decisions remain clinical and safety-driven [3, 16]. Quantitative sIgE trends may support follow-up but should be interpreted within a single platform [17, 19]. 

**Drug allergy**

Serum sIgE is available for selected drugs (notably β-lactams) but shows variable sensitivity and specificity with limited negative predictive value. A negative result does not exclude IgE-mediated drug allergy; drug provocation testing remains the reference where appropriate. Use sIgE selectively within a structured diagnostic algorithm [29, 30]. For biologics and other agents with available assays, interpret results cautiously and in context [29]. 

**Clinical integration**

tIgE and sIgE support but do not define disease. Testing should be hypothesis-driven after an allergy-focused history; indiscriminate panels increase overdiagnosis of sensitization. When discordant with history, consider assay features, CCDs, extract composition, pre-test probability, and for foods, supervised oral food challenge as gold standard. For venoms and drugs, weigh risks and guideline-based pathways for skin and provocation testing; use sIgE to complement – not replace – these steps [1, 2, 16, 29]. 

**Communication and reporting**

Reports should state assay/platform, allergen source (extract vs. component), units, and any factors that may affect interpretation (e.g., CCD interference, low-titer patterns, high tIgE context). Serial testing should remain on a single platform. Where decision points are used, their origin and limitations should be specified [11, 15, 17]. 

## Multiplex assays and screening tests 

Multiplex IgE assays (microarrays/macroarrays) measure IgE against many components simultaneously, providing broad sensitization profiles in polysensitized patients and supporting hypothesis generation when the clinical picture is unclear [1, 4]. Platforms differ in content and analytics: ISAC reports semi-quantitative ISU (ordinal units rather than absolute concentrations) for components; ALEX2 adds extract testing, total IgE normalization, and CCD inhibition [31, 32, 33]. Screening tests (e.g., Phadiatop, Phadiatop Infant; mold mixes such as mx1) provide rule-in/rule-out information for atopy or exposure-related phenotypes and may triage further testing [34, 35, 36]. Indiscriminate use in low pre-test probability settings may lead to overdiagnosis and should be avoided. 

### Indications and clinical scope 

Typical indications include (i) polysensitization where singleplex testing would be impractically broad; (ii) suspected cross-reactivity patterns (PR-10, profilins, polcalcins, nsLTPs, and storage proteins; *Hymenoptera* venom components; selected drug-related markers) to refine risk and guide management; and (iii) pre-AIT profiling when component patterns influence product choice or predict safety [1, 4]. Multiplex is not a substitute for an allergy-focused history or, for foods, supervised challenges; it complements singleplex by efficiently mapping patterns [4, 37]. 

### Evidence base and platform comparisons 

Clinical comparisons show good but not interchangeable agreement between multiplex and singleplex assays for shared components; correlations vary by molecule and allergen group [38, 39, 40]. Head-to-head studies between ISAC and ALEX2 confirm overall agreement, although ALEX2 may yield higher semi-quantitative values and detect additional extract-level sensitizations; CCD inhibition on ALEX2 reduces carbohydrate-driven false positives [32, 33, 41, 42]. Systematic reviews highlight that diagnostic accuracy data are strongest for a subset of food components and more limited elsewhere; specificity tends to exceed sensitivity, underscoring that negative results do not exclude disease in high pre-test settings [37]. Health-technology assessment suggests that multiplex may be useful in complex, difficult-to-diagnose cases, although cost-effectiveness depends on local pathways and test stewardship [43]. 

### Screening tests (triage) 

Phadiatop (inhalant mix) shows high diagnostic precision for atopy in adults; Phadiatop Infant adapts content for early-life exposures and performs well in young children [34, 35]. These assays are suited to primary triage or epidemiology and may reduce unnecessary panels when negative; positives should be followed by targeted singleplex or component testing driven by exposure and symptoms [1]. However, false-negative results may occur in patients with high total IgE or low but clinically relevant specific sensitizations. For damp/mold-related complaints, mx1-specific IgE may support the diagnosis of mold-associated respiratory symptoms in symptomatic individuals [36]. Screening should be used within an algorithm that considers prevalence, exposure, and downstream testing costs [43]. 

### Interpretation principles (see Table 3) 

Semi-quantitative readouts (i.e., ordinal signal intensities rather than absolute concentrations) differ across platforms; values are not interchangeable and should not be trended across platforms, and approaches such as total IgE normalization (adjusting signal intensity relative to total IgE) require platform-specific interpretation [32, 38]. Component patterns stratify risk: storage proteins and nsLTPs often align with systemic reactions; PR-10/profilins more often with pollen-related OAS; polcalcins indicate broad pollen cross-reactivity; venom component patterns (e.g., Api m/Ves v repertoires) support relevant venom identification and immunotherapy selection [4]. For drugs, IgE targets on multiplex are limited and should be interpreted cautiously, with drug challenges or alternative diagnostics per guideline pathways [4]. Negative multiplex in the face of strong history mandates reconsideration of timing (recent reaction), medication effects, extract selection, and the need for singleplex or sometimes cellular assays [1, 4]. 

### Analytical caveats 

Residual CCD effects may occur depending on the platform and should be interpreted accordingly [27, 33]. Report and consider total IgE normalization where implemented but avoid cross-platform numerical comparisons [31, 32]. Content coverage matters: absence of a clinically relevant component on a given array can yield false reassurance [4]. External quality assurance, ISO 15189:2022 accreditation, and internal verification are necessary for reliable reporting [7, 24]. 

### Practical integration 

A two-step workflow is recommended: (1) targeted singleplex testing guided by history; (2) multiplex for pattern mapping when broad questions persist or when distinguishing primary sensitization from cross-reactivity affects management or immunotherapy selection [1, 4]. For aeroallergens and venom, multiplex refines molecular profiles that align with clinical phenotypes and therapy selection; for foods, it may reduce unnecessary oral challenges when high-risk components are strongly positive but does not replace challenges in high-stakes decisions [4, 37]. Results should always be interpreted in the context of exposure, age, comorbidities, and local allergen prevalence. Indiscriminate use outside such algorithms increases the risk of clinically irrelevant findings. 

### Outlook 

Expanded arrays, improved calibration, and embedded CCD control enhance comparability and clinical yield; although cross-platform harmonization remains incomplete [31, 32, 33]. Prospective studies linking multiplex patterns to clinical outcomes (provocation tests, real-world reactions) beyond food allergy are needed [37, 43]. 

## Cellular allergy diagnostic assays 

Cellular allergy diagnostics represent adjunctive approaches for assessing immune reactivity beyond traditional IgE-based testing [44]. These assays evaluate functional immune responses, providing functional information that may support the interpretation of sensitization in selected cases. Key methods include the Basophil Activation Test (BAT), T Cell Activation Test (TAT), Enzyme-Linked ImmunoSpot (ELISpot), and the Lymphocyte Transformation Test (LTT), each with distinct principles, strengths, and limitations. 

### Immediate-type reactions 

The BAT measures activation of basophils upon allergen exposure, typically via flow cytometry using surface markers CD63 and CD203c [45, 46]. CD63 is a lysosomal membrane protein rapidly translocated to the cell surface upon activation, while CD203c is a more stable marker with higher sensitivity and lower background expression [46]. BAT may support the diagnosis of IgE-mediated food allergies, immediate drug hypersensitivity, and *Hymenoptera* venom allergy [47, 48, 49, 50, 51]. Optimal allergen concentrations are typically determined via dose-response curves (e.g., 0.1 – 100 µg/mL), with appropriate positive (anti-IgE) and negative (medium-only) controls essential [46]. Despite high specificity, BAT requires fresh whole blood, specialized equipment, and trained personnel, limiting widespread use [46]. Another limitation is the presence of non-releasing individuals (approximately 5 – 20%), whose basophils do not upregulate activation markers despite adequate stimulation, likely reflecting impaired FcεRI-mediated signaling. Low basophil counts may further limit test performance. Standardization efforts, including international ring trials (e.g., the EU-funded “Allergen-Specific BAT” project), have improved inter-laboratory comparability [48, 52]. Recent data found a high reproducibility in peanut allergy [53]. 

Mast cell activation tests (MAT) have been introduced [45] but are not commonly used and will therefore not be described in detail. 

### Delayed-type reactions 

TATs assess allergen-specific T-cell responses, often using CD154 (CD40L) or intracellular cytokine staining (e.g., IL-4, IFN-γ). They may support the diagnosis of non-IgE-mediated allergies (e.g., drug hypersensitivity) and cases of suspected immune dysregulation. However, TATs require longer incubation times (48 – 72 hours), are technically demanding, and lack standardized protocols [45]. More recent approaches such as activation-induced marker (AIM) assays allow shorter stimulation times (typically 6-24 hours) and have been explored as alternative approaches in recent studies. 

ELISpot detects cytokine-secreting T cells (e.g., IFN-γ, IL-5) after allergen stimulation. It is sensitive and suitable for detecting low-frequency T-cell responses, making it valuable in research and selected clinical cases. However, it is less practical in routine diagnostics due to cost and technical complexity [54, 55]. In drug allergy diagnostics, the ELISpot assay may detect drug-specific T-cell responses, especially when performed shortly after the allergic event, but remains a complementary method [53, 56, 57]. Standardized reference values for defining positive results are lacking. 

The LTT measures T-cell proliferation via thymidine incorporation or flow cytometry. While useful in delayed-type drug hypersensitivity, it suffers from low sensitivity and long turnaround times (up to 7 days), limiting clinical utility [58, 59]. 

**Clinical integration/practical aspects**

A critical challenge lies in interpreting results in clinical context [60]. Cellular activation does not equate to clinical allergy and must be correlated with the history of symptoms upon exposure. Misinterpretation may lead to overdiagnosis and unnecessary avoidance or treatment. BAT may provide clinically relevant functional information in selected settings, including food and drug allergy, although its added value varies depending on the allergen and comparator tests [45, 61, 62, 63]. Overall, cellular assays should be regarded as adjunctive tools in selected, unresolved cases and do not replace established diagnostic pathways. Their clinical impact is mainly in cases with inconclusive conventional diagnostics. 

European national authorities require External Quality Assurance (EQA) of the performance of diagnostic laboratory tests. The BAT-EQA Task Force provided a standard operating protocol and reference materials for the BAT to standardize and enhance its accuracy [52]. In Germany, EQA programs [24] are only available for BAT with bee and wasp venom, and for LTT with PHA, PMA/ Ionomycin, anti-CD3, and anti-CD3/anti-CD28. 

In conclusion, cellular assays provide functional information in allergy diagnosis but require careful validation, standardization, and integration into clinical decision-making. Their role is limited to selected cases where conventional testing is inconclusive. Currently, official German guidelines for drug hypersensitivity [64], venom allergy [65], and food allergy [66] do not recommend cellular tests for routine allergy diagnostic purposes. Cost-effectiveness remains limited due to high technical demands and staffing requirements, although automation and standardization efforts may improve feasibility. 

### Outlook 

Overall, cellular in vitro allergy diagnostics is on track to expand and improve clinical allergy diagnostics through automation, multiplexing, and precision medicine. Recent developments include bin-based automation approaches of BAT [67], fluorescent allergen tetramer staining [68], or the multiplexing of BAT [69] and ELISpot [70]. Other interesting diagnostic approaches include the determination of sIgE, cytokines, chemokines, and microRNAs in dried blood spots or of parameters in small microfluidic chips or patches applied to the skin [71]. 

## Special aspects 

### Monitoring of allergen immunotherapy 

To date, no in vitro method or biomarker has been established to indicate the success or failure of allergen-specific immunotherapy, and clinical assessment remains essential [72]. The following immunological and functional assays are used in clinical trials and for research purposes:

- A higher baseline sIgE/tIgE ratio has been associated with better clinical response, but does not reliably predict treatment outcome [20, 72].
- Increases in allergen-specific IgG4 (and, in some studies, IgG2) are observed during successful immunotherapy and may serve as surrogate markers of immunological tolerance and compliance. However, their correlation with clinical outcomes is variable [72, 73, 74].
- Serum inhibitory activity for IgE (IgE-FAB assay) and related assays assessing IgE-blocking activity (e.g., IgE-FAP or inhibition ELISA) reflect functional IgG effects and may correlate with clinical improvement [72, 75].
- BAT: reduced basophil activation may be observed in responders and reflects desensitization [72, 76].
- Cellular and cytokine assays: Flow cytometry and ELISA can be used to assess changes in regulatory T cells, regulatory B cells, and cytokine profiles (e.g., IL-4, IL-10, IFN-γ), which reflect underlying immunological mechanisms but are not yet standardized for clinical monitoring [72].

### Anaphylaxis, mast cell disorders 

Serum tryptase is a key biomarker for mast cell activation and mast cell disorders and includes α-tryptase (reflecting mast cell burden) and β-tryptase (reflecting mast cell activation) [77]. Within 15 minutes to 3 hours after onset of anaphylaxis, there is an increase in serum tryptase due to mast cell release of β-tryptase that declines within 24 to 48 hours to basal serum tryptase (BST). Elevated BST levels are often associated with hereditary α-tryptasemia (HαT) [78]. HαT is caused by an increased copy number of the TPSAB1 gene, which codes for α-tryptase, and affects approximately 4 – 7% of the population. TPSAB1 genotyping may be discussed in patients with persistently elevated BST (e.g.,  > 8 – 11 ng/mL), although its clinical relevance remains under discussion [79]. This diagnostic test is currently only offered by specialized laboratories. The clinical relevance of HαT remains controversial [80, 81, 82]. 

### Contact allergy 

There is currently no reliable, clinically validated in vitro diagnostic test available for routine analysis of contact allergy. The gold standard for diagnosing allergic contact dermatitis remains the in vivo patch test. While several in vitro approaches have been investigated – including assays using peripheral blood mononuclear cells, monocyte-derived dendritic cells, LTTs, and gene expression profiling – none have achieved sufficient sensitivity, specificity, or standardization for routine clinical use [83]. 

### Oncology 

In oncology, BAT is an emerging adjunctive tool to assess hypersensitivity reactions to biologics and chemotherapy agents, monitor drug tolerance in desensitization, and predict and address the safety of novel anti-cancer IgE-based therapeutics [84]. 

## Outlook and unmet needs 

Broader molecular coverage, integrated CCD control, and improved interpretive algorithms support a more systematic use of multiplex results, particularly when broader sensitization profiles contribute to clinical decision-making. Consequently, multiplex testing should be regarded as a complement to quantitative singleplex assays, with careful interpretation. 

Across humoral and cellular diagnostics, standardization remains an urgent unmet need. The need is even greater for cellular methods. Cellular diagnostics such as BAT and T-cell assays remain complex, labor-intensive, and dependent on expert-level methodological knowledge. Their performance is strongly influenced by pre-analytics, reagent selection, and gating strategies, making them unsuitable for broad routine use as first-level diagnostics in their current form. As a result, cellular tests should continue to be refined primarily within clinical research settings or additional diagnostics with rigorous protocols and controlled conditions. 

Emerging point-of-care and minimally invasive technologies may broaden access to testing but require further validation. While skin testing currently remains a central and cost-effective component of allergy diagnostics, laboratory-based methods are expected to gain importance as standardized molecular reagents, automated workflows, and integrated interpretive tools become more widely available and clinically validated. 

## Authors’ contributions 

Both authors contributed equally to the conception, writing, revision, and final approval of the manuscript. 

## Funding 

No external funding was received for this work. 

## Conflict of interest 

BW has received honorary for educational lectures and/or advisory boards from ALK-Abelló, Biocryst, CSL-Behring, Kalvista, Novartis, Sanofi-Aventis, Takeda, Thermo Fisher Scientific. TB has received research funding and/or material support for scientific projects from Thermo Fisher Scientific and R-Biopharm. 

**Table 1.** Humoral singleplex assays – analytical and clinical matrix.

Assay	Clinical scope (Food/Aero/Venom/Drug)	Key clinical questions	Analytical features (summary)	Interpretation focus	Decision support and monitoring	Laboratory/QA and reporting	Evidence
Total IgE (tIgE)	Contextual covariate across all domains; ABPA: core component	Does atopic background or ABPA context apply?	WHO traceability (11/234); between-method bias persists; method- and age-dependent reference intervals	Not a binary trigger; very low tIgE lowers allergy probability; high tIgE is non-specific (Do not diagnose allergy based on tIgE alone)	ABPA algorithms; baseline parameter before/during therapy	Document platform; verify local reference intervals; participate in EQA	[1, 5, 6, 7, 8, 9, 24, 25]
sIgE to extracts (singleplex)	Core in food/aero/venom; selective in drug	Is sensitization congruent with exposure and phenotype; contribution to AIT planning	High analytical sensitivity (0.10 kU_A_/L); results are not interchangeable across platforms	0.35 kU_A_/L is not universal; low-titer positives require context; CCD interference possible (Avoid interpretation without clinical correlation)	Food: validated decision points can reduce OFCs (platform- and population-specific); aero/venom: pattern plus severity guide AIT	Keep follow-up on one platform; report method/source/units	[1, 11, 12, 13, 14, 15, 17, 18, 19, 26, 27, 28]
sIgE to components (CRD, singleplex)	Add-on in food/aero/venom; selective in drug	Primary sensitization vs. cross-reactivity; risk stratification; AIT refinement	Molecule-specific; availability/standardization vary	Performance is molecule-/cohort-specific; always correlate clinically (Do not infer risk without clinical context)	Food: storage proteins vs. PR-10/LTP; venom: clarify double positivity	Specify component identity/source; maintain platform consistency	[2, 3, 4, 16]

ABPA = allergic bronchopulmonary aspergillosis; AIT = allergen immunotherapy; CRD = component-resolved diagnostics; CCD = cross-reactive carbohydrate determinants; EQA = external quality assessment; RI = reference interval; OFC = oral food challenge; sIgE = specific IgE; tIgE = total IgE.

**Table 2.** Common pitfalls in humoral in vitro assays and how to mitigate them.

**Pitfall**	**Typical signal pattern**	**Possible consequence**	**Mainly affected domains**	**Practical mitigation**	**Confirmatory/follow-up steps**	**Evidence**
Applying literature cut-offs across different platforms	Borderline on system A not equivalent to system B	Mis-stratification; unnecessary avoidance or missed diagnoses	Food, also aero/venom	Use platform-specific thresholds; keep longitudinal testing on one platform; report method, source, units	If switching platforms, re-baseline and avoid cut-offs during transition	[17, 18, 19]
Treating 0.35 kU_A_/L as a universal boundary	“Positive” without clinical plausibility	Overdiagnosis of sensitization	All	Interpret in clinical context (history, exposure, phenotype, tIgE); accept low-level results only when prior probability is high	Observation or OFC for intermediate scenarios in foods; reassess exposure/seasonality for aero	[1, 2, 11]
Over-interpreting isolated low-titer sIgE when prior probability is low	Single weak positives	Unnecessary restrictions or anxiety (overdiagnosis of sensitization)	All	Refine pre-test probability using targeted history; consider watchful waiting	Foods: supervised OFC when appropriate; aero/venom: confirm exposure relevance	[1, 2]
CCD-driven polyreactivity	Broad low-titer polysensitization due to CCDs	False-positive sensitization profiles	Food, aero, venom	Use component testing or CCD inhibition strategies		[26, 27, 28]
Extrapolating food cut-offs to aero/venom/drug contexts	–	Errors in AIT or drug pathways	Aero, venom, drug	Restrict cut-offs to validated contexts	Aero/venom: guideline-based skin testing/AIT pathways; drug: structured drug provocation testing	[12, 13, 14, 16, 29, 30]
Ignoring EQA and reference-interval issues	–	Unrecognized bias; poor comparability	All	Participate in EQA; verify local RIs and method performance; document calibration traceability (WHO 11/234)	Regular proficiency testing; method verification on implementation	[7, 8, 24, 25]
Switching platforms during follow-up	Apparent titer changes due to method bias	Misinterpretation of trends	All	Avoid switching; if unavoidable, re-baseline	Consider parallel testing overlap if feasible	[17, 19]

ABPA = allergic bronchopulmonary aspergillosis; AIT = allergen immunotherapy; sIgE = allergen-specific immunoglobulin E; tIgE = total immunoglobulin E; CCD = cross-reactive carbohydrate determinants; OFC = oral food challenge; EQA = external quality assessment; RI = reference interval; WHO 11/234 = WHO Standard for human serum IgE (NIBSC code 11/234); IgG4 = immunoglobulin G subclass 4.

**Table 3.** Multiplex platforms vs. singleplex: analytical and clinical comparison.

**Platform type**	**Typical analyte count**	**Sample volume (serum)**	**Readout units**	**Analytical/dynamic range**	**Calibration and traceability**	**Content (molecules vs. extracts)**	**Primary clinical use cases**	**Key limitations**	**Evidence**
Microarray, molecule-focused	100 – 300 molecules	20 – 50 µL	Platform-specific semi-quantitative units	Moderate linear range; potential high-IgE saturation—confirm clinically relevant positives by singleplex	Internal/secondary calibrators; not equivalent to kU_A_/L	Predominantly recombinant/natural molecules; extracts uncommon	Complex polysensitization; limited sample volume; pre-AIT molecular phenotyping; research profiling	Semi-quantitative only; platform-specific scales; not suitable for low pre-test probability screening	[1, 4, 32, 37, 38, 85, 86]
Macroarray, mixed extract+molecule	30 – 100 spots	50 – 150 µL	Ordinal or semi-quantitative classes	Narrower range; coarse gradations—confirm management-relevant findings by singleplex	Internal calibrators; not traceable to kU_A_/L; cross-platform non-equivalence	Mix of selected components with common extracts	Broad first-pass overview; triage to focused singleplex testing; pre-AIT profiling	Limited quantitation; fixed content; potential residual CCD effects; risk of overcalling low-level multi-positivity	[27, 31, 32, 33, 41, 42, 43]
Singleplex (reference)	1 per assay	20 – 50 µL per analyte	kU_A_/L (quantitative)	Wide linear range; suited for monitoring and validated decision points	Calibrated to WHO 11/234; method-specific reference intervals and validated cut-offs available	Extracts and individual components available	Confirmation of clinically relevant sensitizations; application of validated decision points; longitudinal monitoring; pre-AIT confirmation	Requires targeted testing; sample and cost scale with breadth; avoid platform switching	[1, 7, 11, 12, 13, 14, 24]

AIT = allergen immunotherapy; CCD = cross-reactive carbohydrate determinants; RI = reference interval; WHO 11/234 = World Health Organization Standard for human serum IgE (NIBSC 11/234).
